# Supplementary material for: Single-cell RNA sequencing of the Strongylocentrotus purpuratus larva reveals the blueprint of major cell types and nervous system of a non-chordate deuterostome
Source: eLife. 2021 Nov 25;10:e70416. doi: 10.7554/eLife.70416 (PMC8683087; doi:10.7554/eLife.70416)
Supplement: Transparent reporting form [file elife-70416-transrepform1.docx]

***eLife’s* transparent reporting form**

We encourage authors to provide detailed information *within their submission* to facilitate the interpretation and replication of experiments. Authors can upload supporting documentation to indicate the use of appropriate reporting guidelines for health-related research (see [EQUATOR Network](http://www.equator-network.org/%20)), life science research (see the [BioSharing Information Resource](https://biosharing.org/)), or the [ARRIVE guidelines](http://www.plosbiology.org/article/info:doi/10.1371/journal.pbio.1000412) for reporting work involving animal research. Where applicable, authors should refer to any relevant reporting standards documents in this form.

If you have any questions, please consult our Journal Policies and/or contact us: [editorial@elifesciences.org](mailto:editorial@elifesciences.org).

**Sample-size estimation**

- You should state whether an appropriate sample size was computed when the study was being designed
- You should state the statistical method of sample size computation and any required assumptions
- If no explicit power analysis was used, you should describe how you decided what sample (replicate) size (number) to use

Please outline where this information can be found within the submission (e.g., sections or figure legends), or explain why this information doesn’t apply to your submission:

In the single cell transcriptomics field there is no fixed sample size as the number of replicates depends on the intrinsic characteristics of the organisms/cells. For instance, previous scRNA-seq related studies on sea urchin have been carried out by using only one replicate per developmental time-point were carried out. In our case we choose to use thousands of 3 dpf larvae resulting from fertilization of gametes coming from 4 different sea urchin individuals (biological replicates) to account for the high degree of genetic polymorphism sea urchin embryos and larvae bear. Furthermore, we used two additional technical replicates sp72_2a, sp72_1a that are libraries sequenced deeper.

Our sample-size strategy is detailed in sub-section 3 of the Materials and Methods section of the manuscript

**Replicates**

- You should report how often each experiment was performed
- You should include a definition of biological versus technical replication
- The data obtained should be provided and sufficient information should be provided to indicate the number of independent biological and/or technical replicates
- If you encountered any outliers, you should describe how these were handled
- Criteria for exclusion/inclusion of data should be clearly stated
- High-throughput sequence data should be uploaded before submission, with a private link for reviewers provided (these are available from both GEO and ArrayExpress)

Please outline where this information can be found within the submission (e.g., sections or figure legends), or explain why this information doesn’t apply to your submission:

For the single cell RNA-Seq experiments, everything is detailed in the above sections.

In situ hybridizations and immunohistochemistry experiments were performed on at least two biological replicates of larvae and at least 50 specimen per experiment were imaged.

Biological replicates of larvae are obtained upon fertilization and growth using gametes from independent adult individuals. Technical replicates are repetition of the experiments using the same biological replicates.

**Statistical reporting**

- Statistical analysis methods should be described and justified
- Raw data should be presented in figures whenever informative to do so (typically when N per group is less than 10)
- For each experiment, you should identify the statistical tests used, exact values of N, definitions of center, methods of multiple test correction, and dispersion and precision measures (e.g., mean, median, SD, SEM, confidence intervals; and, for the major substantive results, a measure of effect size (e.g., Pearson's r, Cohen's d)
- Report exact p-values wherever possible alongside the summary statistics and 95% confidence intervals. These should be reported for all key questions and not only when the p-value is less than 0.05.

Please outline where this information can be found within the submission (e.g., sections or figure legends), or explain why this information doesn’t apply to your submission:

A part from the automatic statistical analysis done by R for the single cell data (details can be found sub-section 3 of the Materials and Methods section of the manuscript, in this paper we do not have any experiment which requires statistical analysis.

(For large datasets, or papers with a very large number of statistical tests, you may upload a single table file with tests, Ns, etc., with reference to sections in the manuscript.)

**Group allocation**

- Indicate how samples were allocated into experimental groups (in the case of clinical studies, please specify allocation to treatment method); if randomization was used, please also state if restricted randomization was applied
- Indicate if masking was used during group allocation, data collection and/or data analysis

Please outline where this information can be found within the submission (e.g., sections or figure legends), or explain why this information doesn’t apply to your submission:

NA

**Additional data files (“source data”)**

- We encourage you to upload relevant additional data files, such as numerical data that are represented as a graph in a figure, or as a summary table
- Where provided, these should be in the most useful format, and they can be uploaded as “Source data” files linked to a main figure or table
- Include model definition files including the full list of parameters used
- Include code used for data analysis (e.g., R, MatLab)
- Avoid stating that data files are “available upon request”

Please indicate the figures or tables for which source data files have been provided:

We have uploaded as supplementary files to the manuscript two excel books containing gene markers per cell type and the total genes expressed lists as well as a list with the primers to synthetize the probes. These supplementary files are cited throughout the manuscript when needed.

We also uploaded mapped reads of scRNA-seq data and the script used to analyze them. These are available on Dryad at the following link:<https://datadryad.org/stash/share/5LVyoM5CeRznsS6fiJmTcKpYeOTDKBxN0bBKp7VcinI>.
